# Supplementary material for: Cocaine’s cerebrovascular vasoconstriction is associated with astrocytic Ca2+ increase in mice
Source: Commun Biol. 2022 Sep 9;5:936. doi: 10.1038/s42003-022-03877-w (PMC9468035; doi:10.1038/s42003-022-03877-w)
Supplement: Supplementary file 2 — Supplementary Information [file 42003_2022_3877_MOESM2_ESM.pdf]

Supplementary Information

Supplementary Figure 1)

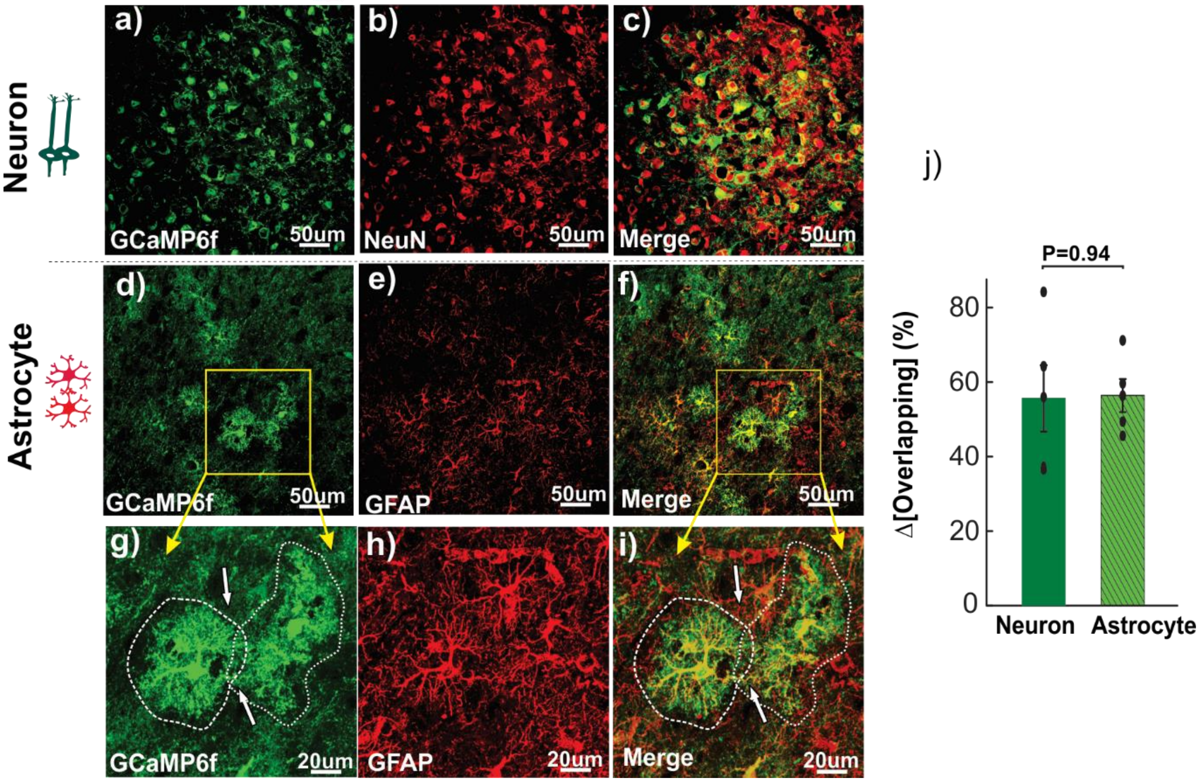

Supplementary Figure 1 (a-c): Ex-vivo fluorescence images of sensorimotor cortex from a wild-type mouse transfected with AAV5-syn-GCaMP6f (a), immunostaining with the antibody to NeuN, which is a neuronal marker (b), and their co-registration for confirmation of GCaMP6f expression in neurons (c). **(d-f):** Ex vivo fluorescence images of sensorimotor cortex from a GFAP-Cre transgenic mouse transfected with AAV5-CAG-Flex-GCaMP6f (d), immunostaining with the antibody to GFAP (e), and their co-registration for confirmation of GCaMP6f expression in glial cells (f). **(g-i):** the high-magnification ex vivo fluorescence images from (d-f), where the arrows show the connection between the astrocytes, and the dash circles show the territory of astrocytes. Statistic comparison of expression overlapping rate between neurons (55.6±8.9%, Group N) and astrocytes groups (56.4 ± 4.4%, Group A) (Supplementary Figure 1j), thus indicating no significant difference of GCaMP6f expression into neurons and astrocytes (n=3 animals/per group, ROIs=5/animal, p=0.94). All error bars are presented as means ± SEM.

Supplementary Figure 2)

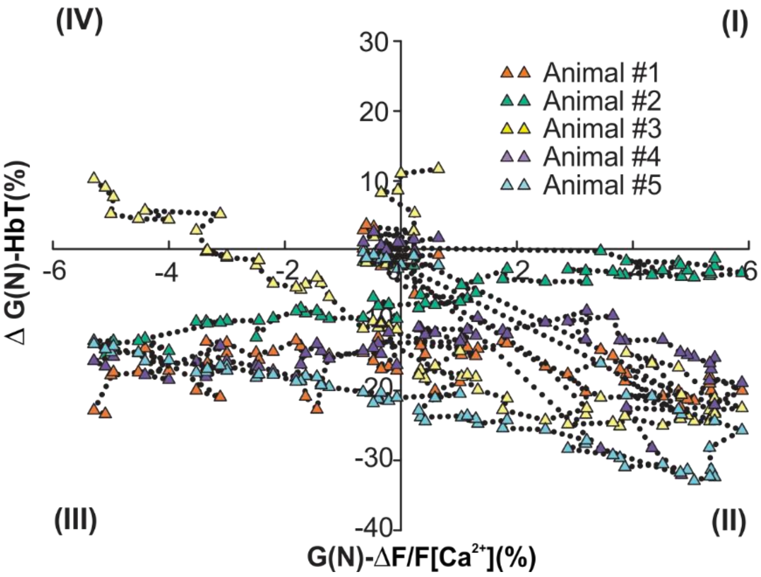

Supplementary Figure 2 Cocaine's effects on neuronal ΔF/FCa^{2+}-G(N) and arterial Δ[HbT]-G(N) changes in the cortex.

Supplementary Figure 2 shows neuronal Ca^{2+} dependent fluorescence changes ΔF/FCa^{2+}-G(N) and blood volume within arteries (Δ[HbT]-G(N)) from t=-10min baseline to t=60min after cocaine, in which the individual animals' responses are presented in different colored dots (n=5). It indicates no direct correlations between neuronal activation and vascular changes in response to cocaine.

Supplementary Figure 3)

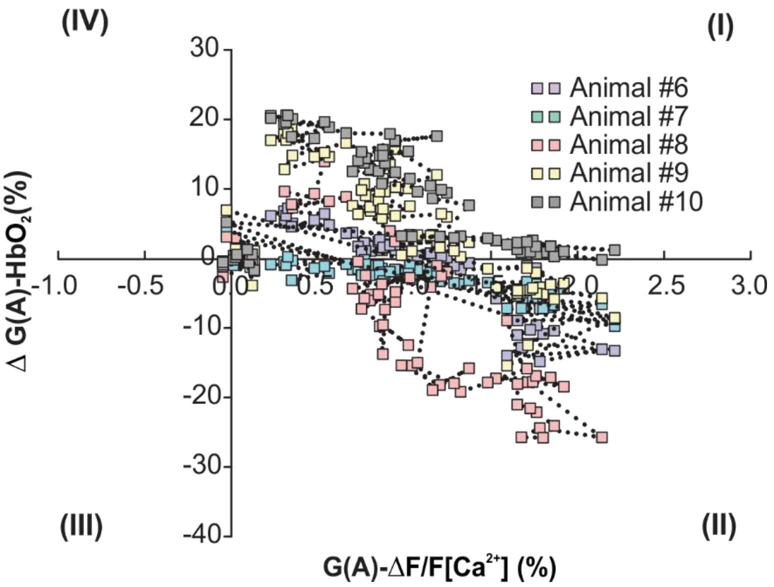

Supplementary Figure 3 Cocaine's effects on astrocytic ΔF/FCa^{2+}-G(A) and tissue Δ[HbO_2]-G(A) changes in the cortex of animals.

Supplementary Figure 3 represents astrocytic Ca^{2+} dependent fluorescence changes ΔF/FCa^{2+}-G(A) along with tissue oxygenated hemoglobin Δ[HbO_2]-G(A) before and after cocaine administration. Each animal's responses are presented by the different colored dots (n=5). Data do not show a linear relationship between ΔF/FCa^{2+}-G(A) and Δ[HbO_2]-G(A).

Supplementary Figure 4)

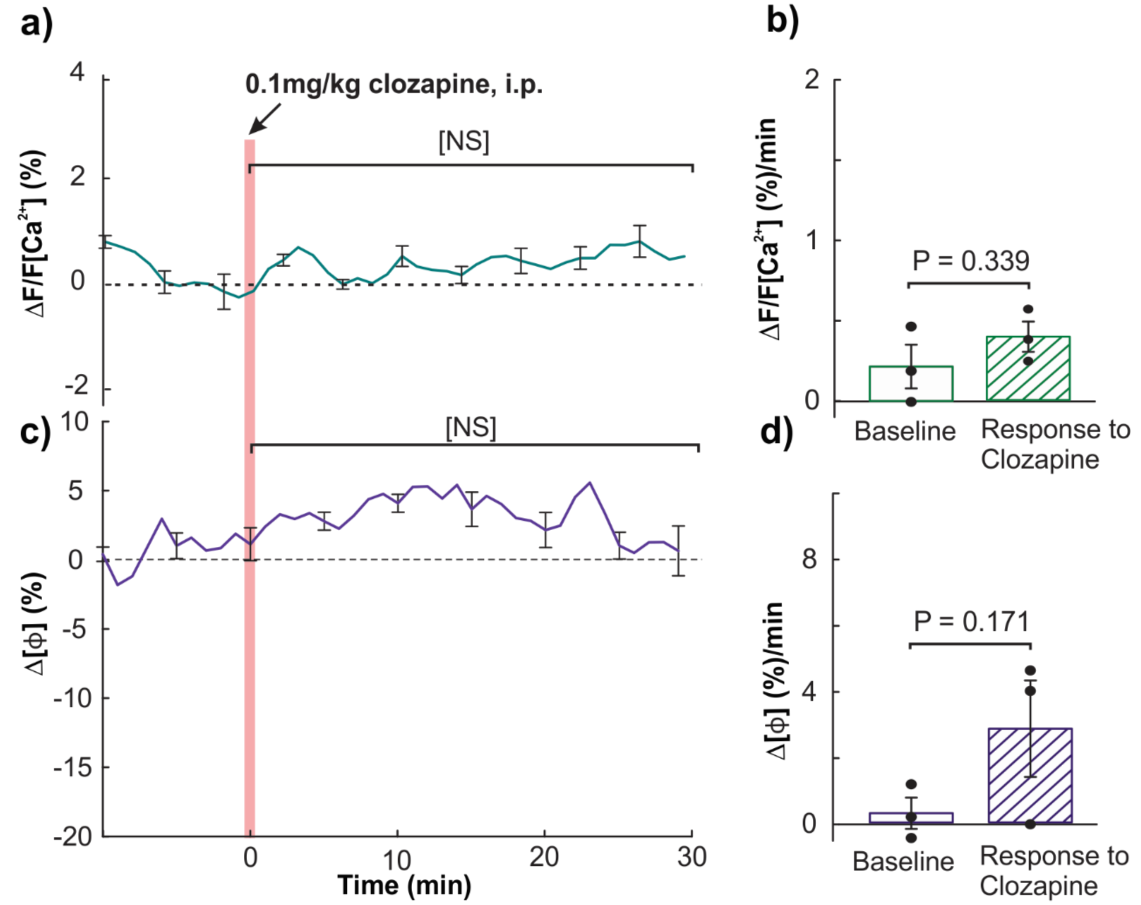

Supplementary Figure 4 Quantification of vessel diameter changes (purple) and neuronal Ca^{2+} dependent fluorescence (green) in response to clozapine.

Supplementary Figure 4 shows the neuronal dependent fluorescence ΔF/F Ca^{2+} and vessel size changes as a function of time in response to clozapine (0.1mg/kg, i.p., n=3). One-way repeated ANOVA showed no significant time effects on neuronal Ca^{2+} after clozapine injection (n = 3,). Meanwhile, quantification analysis of average efficiency in Supplementary Figure 4b shows that there were no differences between before (0.2145 ± 0.1361%) and after (0.3940 ± 0.0940%) clozapine injection (p=0.339). One-way repeated ANOVA showed no significant time effects on vessel diameter size after clozapine injection (n = 3,). Supplementary Figure 4d Quantification analysis of average efficiency shows that no difference before (0.2778 ± 0.4723%) and after (2.829 ± 1.458%) clozapine injection (P=0.171). All error bars are presented as means ± SEM.

Supplementary Figure 5)

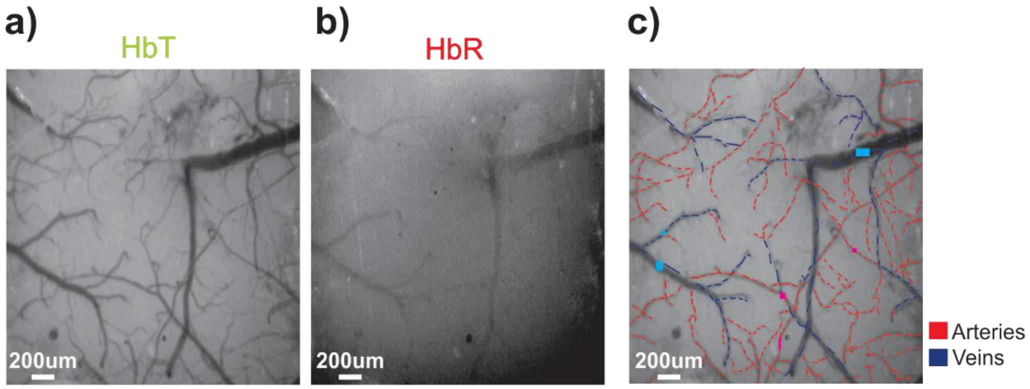

Supplementary Figure 5 Demonstration of separating arteries and veins from HbT and HbR images.

Red traces: arteries; blue traces: veins. As arteries and veins contain predominately oxygenated-hemoglobin (HbO_2) and deoxygenated-hemoglobin (HbR), respectively, the absorbance difference between HbO_2 and HbR at different wavelengths are used to separate arteries and veins. a) At wavelength of 568nm, it is an isosbestic point of HbO_2 and HbR spectra with high absorbance, which is used as HbT channel in our MIP system to detect both arteries and veins. b) In contrast, at wavelength of 630nm, HbR has obviously higher light absorption than $HbO_{2,}$ which is more sensitive to vein as so-called ‘HbR’ channel to distinguish veins. c) Taking the observation from these two wavelengths allowed us to identify arteries and veins.

Supplementary Figure 6)

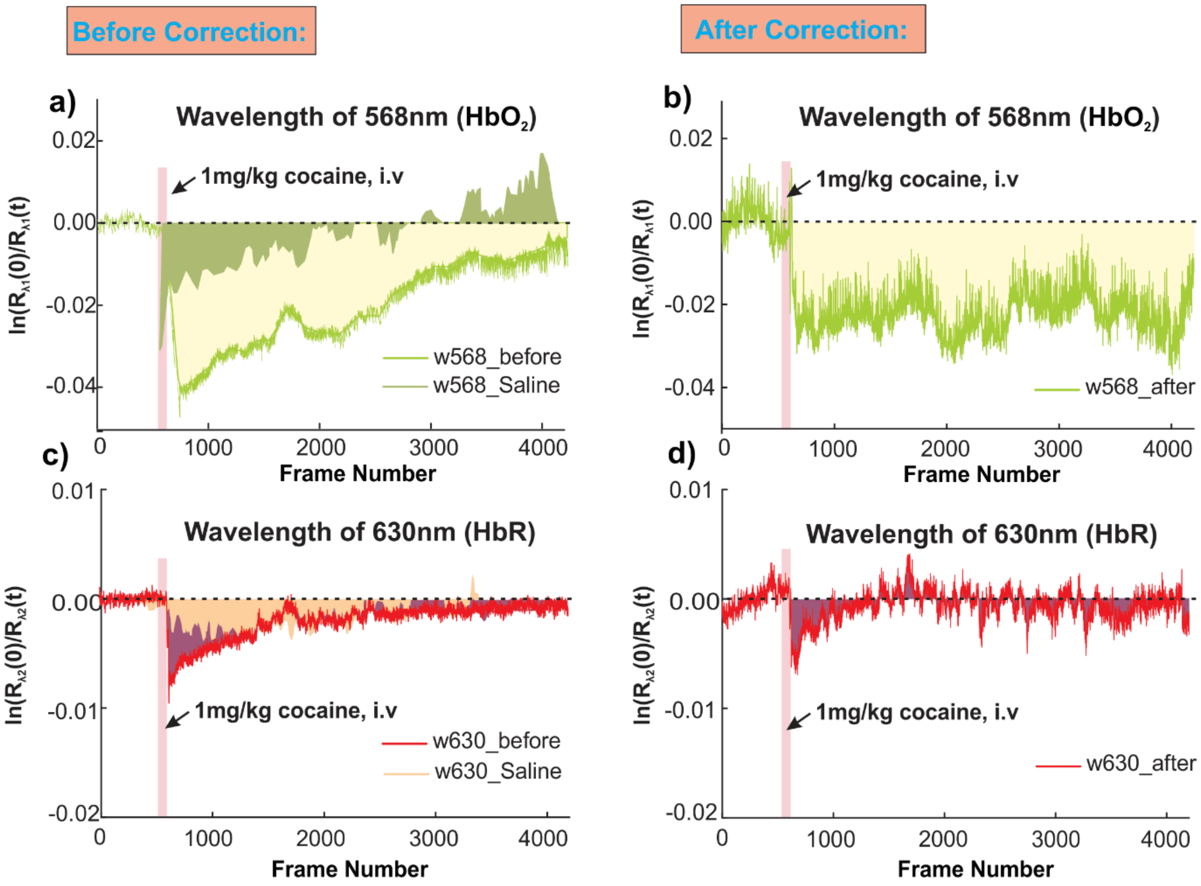

Supplementary Figure 6 Demonstration of correction for the blood volume changes due to injection.

a) Reflectance ($\ln(R_{\lambda1}(0))/R_{\lambda1}(t)$ at wavelength of 568nm in response to cocaine (yellow shadow) or saline (green shadow), respectively. b) Cocaine-induced reflection changes after correction at wavelength of 568nm. c) Reflectance ($\ln(R_{\lambda2}(0))/R_{\lambda2}(t)$ at wavelength of 630nm in response to cocaine (purple shadow) or saline (orange shadow), respectively. d) Similar correction is conducted for HbR channel at wavelength of 630nm.

Supplementary Figure 7)

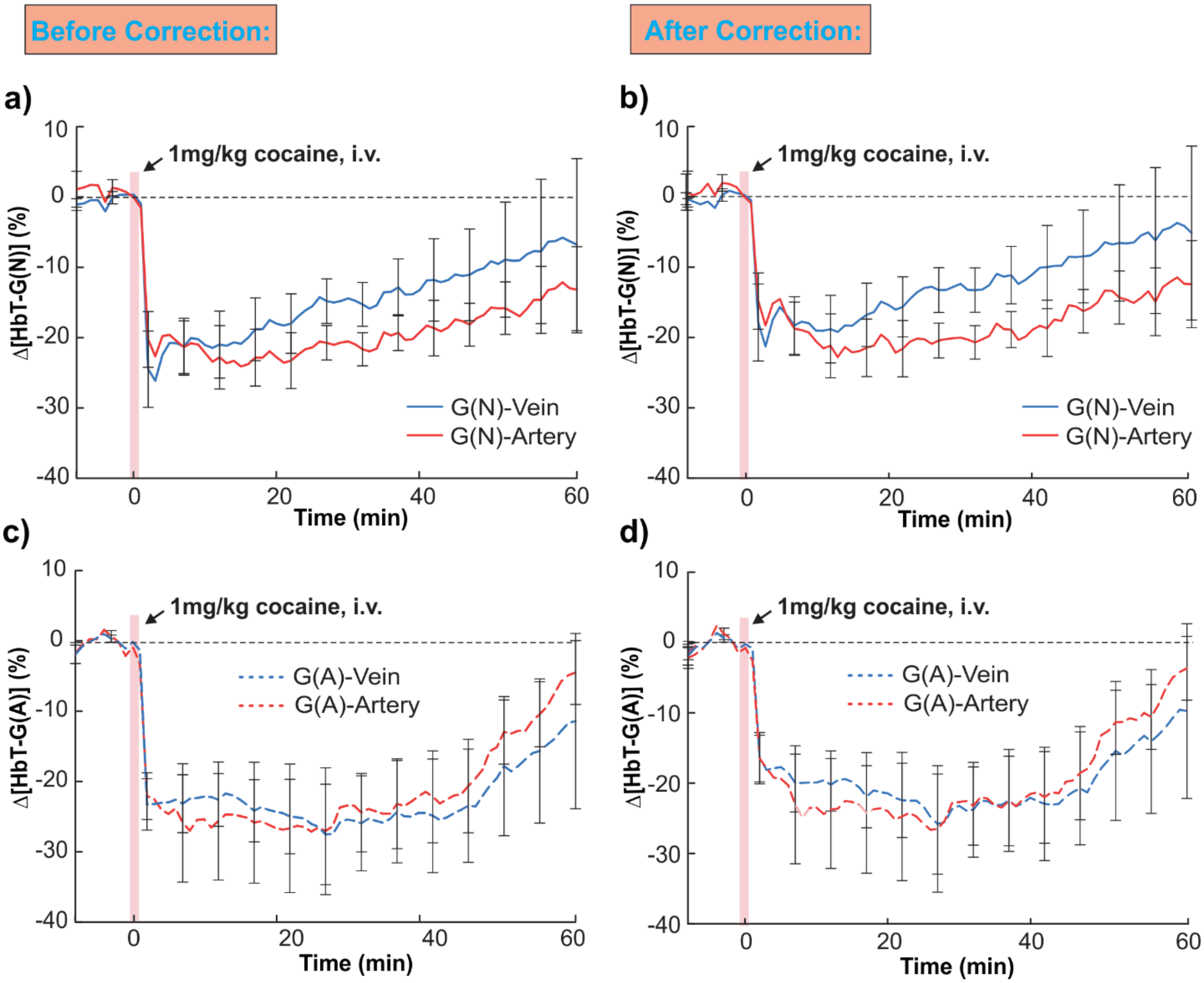

Supplementary Figure 7 Cocaine-induced ΔHbT changes before and after correction.

a) Time courses of Δ[HbT] in arteries (red) and veins (blue) in response to cocaine (1mg/kg, i.v.) in neuronal GCaMP6f-expressed animals (n=5) before correction. b) Time courses of Δ[HbT] in response to cocaine (1mg/kg, i.v.) in neuronal GCaMP6f-expressed animals (n=5) after correction. It indicates the infusion correction slightly reduces ΔHbT changes within injection period (t<2 mins). c) Before correction time course of Δ[HbT] in astrocytic group in response to cocaine. d) Similarly, time courses of Δ[HbT] in response to cocaine (1mg/kg, i.v.) in astrocytic GCaMP6f-expressed animals (n=5) after correction. All error bars are presented as means ± SEM.
