# Supplementary material for: Cocaine’s cerebrovascular vasoconstriction is associated with astrocytic Ca2+ increase in mice
Source: Commun Biol. 2022 Sep 9;5:936. doi: 10.1038/s42003-022-03877-w (PMC9468035; doi:10.1038/s42003-022-03877-w)
Supplement: Supplementary file 3 — Description of Additional Supplementary Files [file 42003_2022_3877_MOESM3_ESM.pdf]

Description of Additional Supplementary Files

File Name: Supplementary Data 1
Description: Source data underlying plots shown in Figures 2-7.
