# Supplementary material for: Cocaine’s cerebrovascular vasoconstriction is associated with astrocytic Ca2+ increase in mice
Source: Commun Biol. 2022 Sep 9;5:936. doi: 10.1038/s42003-022-03877-w (PMC9468035; doi:10.1038/s42003-022-03877-w)
Supplement: Supplementary file 5 — Reporting Summary [file 42003_2022_3877_MOESM5_ESM.pdf]

nature research

Corresponding author(s): Congwu Du COMMSBIO-22-0447B

Last updated by author(s): Jul 29, 2022

Reporting Summary

Nature Research wishes to improve the reproducibility of the work that we publish. This form provides structure for consistency and transparency in reporting. For further information on Nature Research policies, see our Editorial Policies and the Editorial Policy Checklist.

Statistics

For all statistical analyses, confirm that the following items are present in the figure legend, table legend, main text, or Methods section.

n/a	Confirmed	
☐	☒	The exact sample size (n) for each experimental group/condition, given as a discrete number and unit of measurement
☐	☒	A statement on whether measurements were taken from distinct samples or whether the same sample was measured repeatedly
☐	☒	The statistical test(s) used AND whether they are one- or two-sided<br>*Only common tests should be described solely by name; describe more complex techniques in the Methods section.*
☐	☒	A description of all covariates tested
☐	☒	A description of any assumptions or corrections, such as tests of normality and adjustment for multiple comparisons
☐	☒	A full description of the statistical parameters including central tendency (e.g. means) or other basic estimates (e.g. regression coefficient) AND variation (e.g. standard deviation) or associated estimates of uncertainty (e.g. confidence intervals)
☐	☒	For null hypothesis testing, the test statistic (e.g. F, t, r) with confidence intervals, effect sizes, degrees of freedom and P value noted<br>*Give P values as exact values whenever suitable.*
☒	☐	For Bayesian analysis, information on the choice of priors and Markov chain Monte Carlo settings
☒	☐	For hierarchical and complex designs, identification of the appropriate level for tests and full reporting of outcomes
☐	☒	Estimates of effect sizes (e.g. Cohen's d, Pearson's r), indicating how they were calculated

Our web collection on statistics for biologists contains articles on many of the points above.

Software and code

Policy information about availability of computer code

Data collection	Using the program which belong to camera company to record imaging data.
Data analysis	Using ImageJ to sepearte and combin different image channel and a custom Matlab program to obatin vessel sizes quantification.

For manuscripts utilizing custom algorithms or software that are central to the research but not yet described in published literature, software must be made available to editors and reviewers. We strongly encourage code deposition in a community repository (e.g. GitHub). See the Nature Research guidelines for submitting code & software for further information.

Data

Policy information about availability of data

All manuscripts must include a data availability statement. This statement should provide the following information, where applicable:

- Accession codes, unique identifiers, or web links for publicly available datasets
- A list of figures that have associated raw data
- A description of any restrictions on data availability

The datasets generated and/or analysed during the current study are not publicly available, but are available from the corresponding author on reasonable request. There are no datasets from third party included in this paper. Source data underlying plots shown in figures 2-7 provided in Supplementary Data.

Field-specific reporting

Please select the one below that is the best fit for your research. If you are not sure, read the appropriate sections before making your selection.

☒ Life sciences ☐ Behavioural & social sciences ☐ Ecological, evolutionary & environmental sciences

For a reference copy of the document with all sections, see nature.com/documents/nr-reporting-summary-flat.pdf

Life sciences study design

All studies must disclose on these points even when the disclosure is negative.

Sample size	Sample size was estimated based on previous paper (2018 Jun 26;23(13):3878-3890. doi: 10.1016/j.celrep.2018.05.091) and our preliminary result. Sample size in each experiment are indicated in each figure legend and summarized in Table 1.
Data exclusions	All recorded data was used.
Replication	The experimental results and findings can be reproducible.
Randomization	All animals were randomly assigned in each group.
Blinding	Investigators were not blinded during experiments.

Reporting for specific materials, systems and methods

We require information from authors about some types of materials, experimental systems and methods used in many studies. Here, indicate whether each material, system or method listed is relevant to your study. If you are not sure if a list item applies to your research, read the appropriate section before selecting a response.

Materials & experimental systems

n/a	Involved in the study	
☐	☒	Antibodies
☒	☐	Eukaryotic cell lines
☒	☐	Palaeontology and archaeology
☐	☒	Animals and other organisms
☒	☐	Human research participants
☒	☐	Clinical data
☒	☐	Dual use research of concern

Methods

n/a	Involved in the study	
☒	☐	ChIP-seq
☒	☐	Flow cytometry
☒	☐	MRI-based neuroimaging

Antibodies

Antibodies used	Primary antibodies: chicken anti-GFP antibody, mouse anti-Neun antibody, and rabbit anti-GFAP antibody.<br>Secondary antibodies: Alexa Fluor 488 donkey anti-chicken, Alexa Fluor 594 donkey anti-mouse, and Alexa Fluor 594 donkey anti-rabbit.
Validation	The primary antibody chicken anti-GFP antibody(https://www.thermofisher.com/antibody/product/GFP-Antibody-Polyclonal/A10262) was uesd to label viruses. The primary antibody mouse anti-Neun (https://www.emdmillipore.com/US/en/product/Anti-NeuN-Antibody-clone-A60,MM_NF-MAB377) was used to label neuron cells. The primary antibody rabbit anti-GFAP (https://www.sigmaaldrich.com/US/en/product/sigma/g92690) was used to label astrocyte cells.

Animals and other organisms

Policy information about studies involving animals; ARRIVE guidelines recommended for reporting animal research

Laboratory animals	Transgenic B6.Cg-Tg(Gfap-cre)77.6Mvs/2J mice (Jackson Laboratory, https://www.jax.org/strain/024098) and C57BL/6 wild type (WT) mice (Jackson Laboratory) were used for this study when they reached ~8 weeks of age.
Wild animals	N/A
Field-collected samples	N/A
Ethics oversight	All experimental procedures were approved by the Institutional Animal Care and Use Committee at Stony Brook University and conducted according to the National Institutes of Health (NIH) Guidelines for Care and Use of Laboratory Animals.

Note that full information on the approval of the study protocol must also be provided in the manuscript.
